# Intervention-Induced Changes in Balance and Task-Dependent Neural Activity in Adults with Acquired Brain Injury: A Pilot Randomized Control Trial

**DOI:** 10.3390/s24134047

**Published:** 2024-06-21

**Authors:** Jesus A. Hernandez-Sarabia, Arlene A. Schmid, Julia L. Sharp, Jaclyn A. Stephens

**Affiliations:** 1 Department of Health and Exercise Science, Colorado State University, Fort Collins, CO 80523, USA; 2 Department of Occupational Therapy, Colorado State University, Fort Collins, CO 80523, USA; arlene.schmid@colostate.edu; 3 Columbine Health Systems Center for Healthy Aging, Colorado State University, Fort Collins, CO 80523, USA; 4 Sharp Analytics, LLC, Fort Collins, CO 80524, USA; julia.l.sharp@gmail.com; 5 Molecular Cellular, and Integrative Neuroscience Program, Colorado State University, Fort Collins, CO 80523, USA

**Keywords:** neural activity, traumatic brain injury, stroke, functional near-infrared spectroscopy, balance, postural control, randomized controlled trial intervention

## Abstract

Advances in neuroimaging technology, like functional near-infrared spectroscopy (fNIRS), support the evaluation of task-dependent brain activity during functional tasks, like balance, in healthy and clinical populations. To date, there have been no studies examining how interventions, like yoga, impact task-dependent brain activity in adults with chronic acquired brain injury (ABI). This pilot study compared eight weeks of group yoga (active) to group exercise (control) on balance and task-dependent neural activity outcomes. Twenty-three participants were randomized to yoga (n = 13) or exercise groups (n = 10). Neuroimaging and balance performance data were collected simultaneously using a force plate and mobile fNIRS device before and after interventions. Linear mixed-effects models were used to evaluate the effect of time, time x group interactions, and simple (i.e., within-group) effects. Regardless of group, all participants had significant balance improvements after the interventions. Additionally, regardless of group, there were significant changes in task-dependent neural activity, as well as distinct changes in neural activity within each group. In summary, using advances in sensor technology, we were able to demonstrate preliminary evidence of intervention-induced changes in balance and neural activity in adults with ABI. These preliminary results may provide an important foundation for future neurorehabilitation studies that leverage neuroimaging methods, like fNIRS.

## 1. Introduction

Acquired brain injuries (ABIs) are not hereditary or congenital [1], but rather, result from internal processes or exposure to external forces after birth. Internal processes include strokes, infections, tumors, or anoxia. External forces are those that impact the head, trunk, or body—resulting in rapid acceleration and deceleration of the brain within the skull, eliciting traumatic brain injuries (TBIs) [2]. Strokes and TBIs are the leading causes of ABI, affecting approximately two million people in the United States each year [3,4], and may result in long-term cognitive, emotional, and physical impairments [5,6,7]. Two long-term consequences of ABI may be decreased brain function [8] and balance impairments [9]. Notably, balance impairment may be a result of decreased brain function and physical changes in movement, strength, and range of motion (i.e., paresis), leading to postural instability. In those with ABI, physical activity interventions have been used to improve postural stability during balance [10]. Hatha yoga is a physical activity intervention used to improve balance in adults with chronic stroke [11], TBI [12], and ABI [13]. For example, Stephens and colleagues [12] reported that eight weeks of hatha yoga twice a week improved balance in adults with chronic ABI. Importantly, yoga may not only improve balance, but it may also improve brain structure and function, as shown in healthy adults [14]. For example, compared to control participants, experienced yoga participants exhibit less task-dependent activity in the left dorsolateral prefrontal cortex during a Sternberg working memory task; the authors suggest that these findings may represent increased neural efficiency [15]. Thus, it is possible that yoga will impact the brain function and balance of adults with chronic ABI.

However, most of what we know about brain structures [16] and function [17] associated with balance performance is from neuroimaging methods (e.g., functional magnetic resonance imaging [fMRI] and positron emission tomography [PET]) that—with the exception of two studies using PET during standing [18,19]—either simulate balance or require participants to imagine balance tasks while in a supine position during the brain imaging scan. More recently, advances in neuroimaging technology, like functional near-infrared spectroscopy (fNIRS), allow for examination of cerebral cortex neural activity during functional movements, like balance, without restricting individuals in a scanner. Specifically, fNIRS supports measurements of task-dependent neural activity during functional movements in more naturalistic environments [20]. By emitting near-infrared (NIR) light through the scalp and detecting refracted light, fNIRS is able to quantify changes in oxygenated (HbO) and deoxygenated (HbR) hemoglobin based on their NIR light absorption properties [20,21]. These changes in HbO and HbR are indirect measurements of brain activity [22]. As such, fNIRS is an excellent technique for measuring the neural underpinnings of functional capacities, like balance, after interventions meant to improve such functional capacities. Importantly, our team has already established the feasibility of using fNIRS before and after a hatha yoga intervention [23].

A recent review paper [24] highlighted the paucity of literature measuring the neural underpinnings, or task-dependent neural activity, of balance using fNIRS in healthy populations (n = 12 studies) and adults with brain injury (n = 3 studies). Two of the three studies from this review that included participants with brain injury were cross-sectional designs that did not include an intervention (e.g., [25,26]). Considering that improvements in balance have been shown in individuals with ABI after eight weeks of structured training (e.g., hatha yoga), there is a need to examine the neural underpinnings of balance after such interventions in ABI. To date, there appears to be only one study that has evaluated how an intervention (i.e., a combination of physical and occupational therapy) changed task-dependent neural activity during balance using fNIRS in adults with stroke [27]. After the intervention, there was increased HbO in the supplementary motor area (SMA) during anterior-posterior perturbed balance conditions, as compared to HbO levels before the intervention [27]. The authors concluded that the SMA may play a role in balance, and additional studies—such as those that use brain stimulation or neurofeedback—could further elucidate the role of the SMA. Still—to our knowledge—there are no previous studies that have used fNIRS to examine changes in brain activity during balance before and after a yoga intervention in adults with ABI.

Despite limited previous research, it appears that yoga could improve the brain function of adults with chronic ABI. Additionally, previous research has found that yoga can improve balance in adults with ABI [11,12,13], but it is unclear whether yoga provides unique benefits or if comparable exercises, like a low-impact exercise [28], offer similar benefits to balance performance. Thus, the primary purpose of this study is to compare the impact of eight weeks of adaptive yoga with low-impact exercise on balance and task-dependent neural activity in adults with chronic ABI. The primary hypothesis is that both adaptive yoga and low-impact exercise will elicit balance improvements and changes in task-dependent neural activity. Additionally, given previous findings from Fujimoto et al. [27], we expect to find increased HbO activity in the SMA post-intervention.

## 2. Materials and Methods

### 2.1. Study Design

A detailed report of all study procedures has been provided in a study protocol manuscript, Stephens et al. [29]. As such, a focused description of pertinent study methods, following CONSORT reporting guidelines, is included here. This study was a single-blind, randomized controlled trial, and all study procedures were approved by the Institutional Review Board at Colorado State University. All study participants were randomly assigned to an intervention: adaptive yoga group or low-impact exercise group. For brevity, these interventions will be referred to as ‘yoga’ and ‘exercise’ throughout the manuscript. Participants were assessed by masked assessors at a pre-intervention visit and post-intervention visit, which occurred two to three weeks before the first intervention session and within two weeks of the final intervention session, respectively. Each intervention included one-hour class sessions that occurred twice/week for eight total weeks (i.e., 16 h).

### 2.2. Participants

Participants with chronic ABI were recruited for this study via posted flyers, radio and newspaper ads, and communication with local brain injury providers and community organizations who shared study information using list servers, quarterly newsletters, and support groups. Participants were included if they were at least 18 years old, had sustained an ABI at least six months before the start of the study, and had self-reported moderate or greater balance issues, as assessed with the Neurobehavioral Symptom Inventory [30]. Participants were excluded if their most recent ABI occurred less than six months before the start of the study or if they self-reported mild balance impairment or no balance impairment. Additionally, participants were screened for magnetic resonance imaging (MRI) safety using standard criteria. Only participants who met the safety criteria completed the MRI portions of the study, but they were not excluded from the study if they were ineligible for MRI. Participant incentives included an exercise mat, a smartwatch, and a heart rate monitor. These items were used throughout both interventions, so participants received their incentives after the final intervention session.

### 2.3. Group Allocation

Prospective participants were screened over the phone to assess eligibility. Those meeting the inclusion criteria were enrolled in the study and scheduled for a pre-intervention assessment visit where they provided informed written consent and then completed outcome measures. Additionally, after completing outcome measures at the pre-intervention visit, participants were informed of their group allocation by receiving a sealed envelope with their group assignment and necessary information (i.e., location, time, parking, and appropriate attire). Group allocation was determined using a random allocation sequence created by the study biostatistician using the pseudo-random number generator function rand () in Microsoft Excel. The pseudo-random number generated assigned Subject ID numbers into either yoga or exercise. Participants’ Subject ID numbers were assigned using the order of their pre-intervention visits. For example, the participant who attended the first pre-intervention assessment visit was given a Subject ID of ‘001.’ Although the study assessors were aware of participants’ Subject ID, they were unaware of which Subject ID numbers had been assigned to which groups. Subsequently, the assessors were able to provide participants with sealed envelopes—prepared by an unmasked study team member—that included a Subject ID on the exterior and their group assignment inside the envelope.

### 2.4. Outcome Measures

Participants completed outcome measures with masked assessors at pre-intervention and post-intervention assessment visits. A complete list of all outcome measures is included in Stephens et al. [29], and the outcome measures used to generate this study’s findings are described in detail.

#### 2.4.1. Anatomical Brain Scans

Anatomical, functional connectivity, and structural connectivity data were acquired using a 3T MRI System (Siemens MAGNETOM Skyra) with a 64-channel radiofrequency coil, but only anatomical brain scans were used for this study. Thus, T1- and T2-weighted anatomical scans were visually inspected, and T2-weighted scans from pre-intervention visits were used to check for lesions, as lesions could have influenced fNIRS findings. To obtain T1-weighted anatomical scans, a 3D T1-weighted magnetization-prepared rapid gradient-echo pulse sequence was used. To reduce acquisition time, a generalized autocalibrating partially parallel acquisition with the following parameters was used: repetition time (TR) = 2400 ms; inversion time (TI) = 1000 ms; time to echo (TE) = 2.32 ms; flip angle = 8°; field of view (FOV) = 230 mm × 230 mm; matrix size = 255 × 255; in-plane resolution = 0.9 mm; slice thickness = 0.9 mm; slices = 192; slice spacing = 0; and acceleration factor = 2; acquisition orientation = sagittal. To obtain T2-weighted anatomical scans, a fluid-attenuated inversion recovery sequence with the following parameters was used: 0.9 mm^2^ resolution, 192 slices, FOV read = 230 mm, TR = 5000 ms, TE = 387 ms, and TI = 1800 ms.

#### 2.4.2. Balance Performance and Task-Dependent Neural Activity

##### Measures

**Figure 1:**
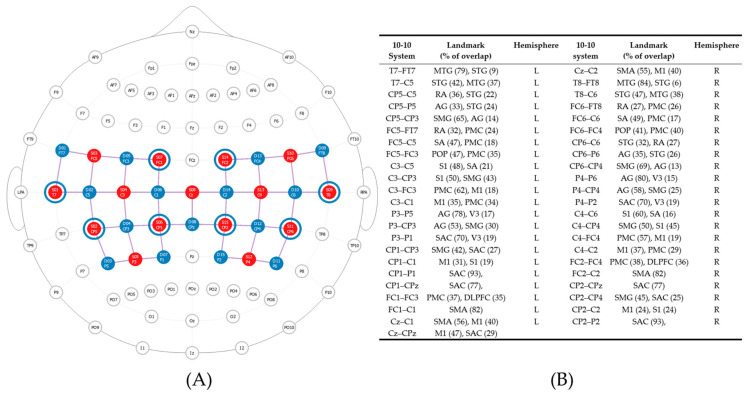
FNIRS head probe. (**A**) Functional near-infrared spectroscopy head probe and anatomic landmarks. This head probe included 15 light sources (red dots) and 15 light detectors (blue dots), which created 45 source–detector pairs, or channels (purple lines), over multiple ROIs. Additionally, eight short separator channels (blue rings around red dots) were included to measure scalp perfusion; (**B**) anatomical landmarks were based on the 10-10 standard EEG system. Each source–detector pair, or channel, was situated over 10-10 EEG coordinates (e.g., T7–FT7). The associated brain area (anatomical landmarks) with specificity (% of overlap) of these channels was defined using the Brodmann Atlas from fNIRS Optodes’ Location Decider [34]. Abbreviations: AG: Angular Gyrus, DLPFC: Dorsolateral Prefrontal Cortex, MTG: Middle Temporal Gyrus, M1: Primary Motor Cortex, PMC Premotor Cortex, POP: Pars Opercularis, RA: Retrosubicular Area, SA: Subcentral Area, SAC: Somatosensory Association Cortex, SMA: Supplementary Motor Area, SMG: Supramarginal Gyrus, STG: Superior Temporal Gyrus, S1: Primary Somatosensory Cortex, V3: Visual Area Three. L: Left, and R: Right.

To assess balance performance and task-dependent neural activity, data were collected simultaneously using a force plate and an fNIRS device at both pre-intervention and post-intervention assessment visits. Balance trials included: bipedal standing on a firm surface with eyes open (EO_Firm_); bipedal standing on a firm surface with eyes closed (EC_Firm_); bipedal standing on a soft surface (foam pad) with eyes open (EO_Soft_); and bipedal standing on a soft surface with eyes closed (EC_Soft_). A previously validated force plate device (Balance Tracking Systems, Inc., San Diego, CA, USA) [31,32,33] was used to record the center of pressure (COP) excursion (path length) during each balance trial. The COP excursion was recorded at a sampling rate of 25 Hz using BTrackS Explore Balance v2.0.4 software. Additionally, a portable fNIRS device, the NIRSport2 (NIRx Medical Technologies, LCC, Berlin, Germany), was used to measure task-dependent neural activity via indirect proxy measures of HbO and HbR during each balance trial. Task-dependent neural activity was recorded with a sampling rate of 4.7 Hz using Aurora software (v2021.9, NIRx Medical Technologies, LCC, Berlin, Germany). This NIRSport2 is a portable system with a limited number of optodes, so regions of interest (ROIs) were determined a priori from the literature. These ROIs—the bilateral inferior parietal and bilateral primary motor cortices and the bilateral supplementary motor area—that support postural control during balance [16,27] were evaluated, along with other nearby ROIs; see Figure 1 for a complete list. A head probe with 30 optodes (15 light sources and 15 light detectors) was designed using the fNIRS Optodes Location Decider (fOLD) toolbox [34] to create 45 total channels over the ROIs. Additionally, eight short separator channels were placed in the cap to measure scalp perfusion [35,36]; see Figure 1.

##### Procedure

**Figure 2:**
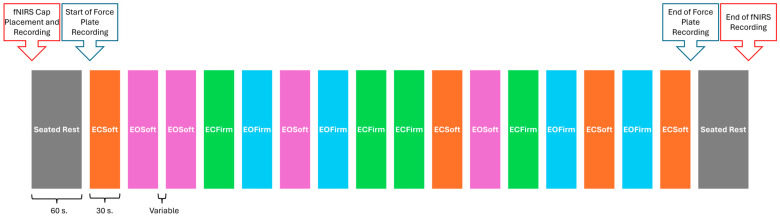
Block design. Visual depiction of randomized block design used to administer balance trials during simultaneous fNIRS recording.

First, we measured participants’ head circumferences and donned an appropriately sized fNIRS cap. Then, fNIRS signal optimization was completed using Aurora software (v2021.9, NIRx Medical Technologies, LCC, Berlin, Germany). As necessary, a shower cap was placed over the fNIRS cap to reduce inference from overhead fluorescent lights and to improve signal quality. After signal optimization, baseline neural activity data were acquired during one minute of seated rest. Then, the balance assessment began. Each balance trial was 30 s in duration with variable inter-trial intervals (ITIs). During each ITI, participants were allowed to rest, as needed, and were given verbal instructions for the next balance trial. To generate an average neural response, balance trials were repeated four times, totaling 16 total trials per participant, using a randomized block design to prevent neural habituation; see Figure 2. Although the order of balance trials was randomized, trials were presented in the same random order for each participant. The order of balance trials was displayed on a computer monitor using PsychoPy software (v.2012.1.3) [37,38,39], and the assessor (who was positioned next to the computer monitor) gave instructions to participants for each balance trial. PsychoPy software also generated event markers in the fNIRS data file via a lab streaming layer. For all balance trials, participants were instructed to stand on the force plate with their arms crossed and feet shoulder-width apart and equidistant from the center of the force plate. As needed, participants were allowed to rest in a chair between balance trials. Following completion of all 16 balance trials, participants completed another minute of seated rest, and then the fNIRS cap was removed. In total, this procedure took between 15 and 30 min.

#### 2.4.3. Force Plate Balance Data Processing

Force plate data were manually exported and analyzed offline using a custom-made script in Matlab 2023a (The MathWorks, Inc., Natick, MA, USA). Specifically, the Matlab script calculated the center of pressure length (COP_Length_) [40,41], the anterior-posterior range (AP_Range_), and the medio-lateral range (ML_Range_) [41] from seconds 5 to 30 of each trial. The first five seconds of each condition were excluded for balance measurements to allow for five seconds of adaptation to each posture, as previously implemented by Richmond and colleagues [33]. After excluding trials in which participants required physical support to avoid falling, an average COP_Length_, average AP_Range_, and average ML_Range_ was calculated for each condition (EO_Firm_, EC_Firm_, EO_Soft_, and EC_Soft_) and visit (pre- and post-intervention) per participant. Larger COP_Length_, AP_Range_, and ML_Range_ values indicate more postural sway, or instability, while standing.

#### 2.4.4. fNIRS Task-Dependent Neural Activity Data Processing

##### fNIRS Processing Steps

FNIRS data were processed offline with Satori (v2.0, NIRx Medical Technologies, LCC, Berlin, Germany). After data files were uploaded into Satori, the raw signal was automatically converted to optical density and then to HbO, HbR, and total hemoglobin values using the Modified Berr–Lambert Law [42]. Then, numbered event markers were manually renamed with the name of each balance condition (e.g., Event 1 = “EC_Soft_”). Next, spikes within the signal were removed by applying a spike-removal monotonic interpolation using Satori default parameters (10 iterations, 5 s lag, 3.5 threshold, and 0.5 influence). Then, high-frequency bands were restored using Temporal Derivative Distribution Repair (TDDR) [43], and physiological noise was removed using a Gaussian low-pass smoothing filter and a Butterworth high-pass filter with cut-off frequencies of 0.4 Hz and 0.01 Hz, respectively. A generalized linear model (GLM) based short-channel regression (SSR) was completed using the highest correlated short channel to detect artifacts and eliminate non-hemodynamic response components from the signal. Data normalization was completed using the Z-transform function, so that data could be compared between subjects. Finally, normalized beta coefficients were generated using the GLM function for HbO, and HbR, per channel and condition for each participant.

Results include changes in HbO and HbR task-dependent neural activity. However, the Discussion will exclusively focus on HbO changes since HbO may be more sensitive to change in task-dependent neural activity [44].

##### fNIRS Data Quality

All previously described data processing steps were performed twice, once without automated channel rejection and once with automated channel rejection. In Satori, automated channel rejection uses a default scalp coupling index (SCI) threshold at <0.75 as an indicator of good data quality [45]. When the automated channel rejection step is completed, it generates a channel rejection map which indicates which channels were automatically rejected, based on their SCI value. Although data that were processed without automated channel rejection were used for analysis, the channel rejection map was used, along with outlier data (defined as Z-scores ≤ −3 or ≥3), when reviewing significant findings to ensure that channels with exceptionally low SCI values were not unduly influencing fNIRS findings. Finally, 74% of participants were eligible for MRI, so their anatomical scans were visually inspected with Mango Software (v4.1, Research Imaging Institute, UTHSCSA) to check for superficial cortical lesions over the fNIRS’ ROIs, and it was also used, as necessary, to exclude participants with significant lesions from fNIRS analyses.

### 2.5. Study Intervention

Participants attended hour-long sessions of yoga or exercise twice a week for eight weeks. For both groups, sessions were held at the same time and day of the week within the same building on a college campus. Interventionists delivering yoga and exercise interventions were required to be trained and certified instructors. Additionally, both interventionists followed standardized protocols that were previously designed and validated [12,13,28].

Yoga sessions included various activities such as breath work (pranayama), stretching and holding of postures (asanas) paired with breath, mantra (repeated words), and meditation (dhyana). During each session, the interventionist provided guidance and adaptations by modifying poses, providing hands-on assistance, and using chairs to support appropriate completion of postures.

Exercise sessions were designed to match the estimated metabolic cost of the yoga group, which was 2.5 Metabolic Equivalent of Task (MET) [46]. Each session included a ten-minute warm-up, an approximately forty-minute exercise program with five stations, followed by a ten-minute cool-down. The five exercise stations included: (1) walking in place, (2) seated exercise (e.g., toe touches), (3) resistance band exercises (e.g., biceps curl), (4) weight-bearing exercise (e.g., squats), and (5) core workout (e.g., torso twists). To ensure that participants maintained a low workout intensity, defined as 2.0 to 3.0 MET or 30 to 40% heart rate reserve, the interventionists calculated the heart rate zones of each participant and monitored their intensity with a smartwatch: the Polar Unite watch (Polar Electro Oy, Kempele, Finland). They also assessed participants’ rate of perceived exertion (RPE = one to three) during each session by showing a modified Borg Scale [47] table and asking participants to rate their perceived exertion on a scale from zero to ten (maximal effort).

### 2.6. Statistical Analysis

Participants who attended at least seven intervention sessions were included in data analysis, which was completed using IBM SPSS Statistics (v 29.0.0.0). This dosage amount of seven sessions was determined using feasibility study data [23]. First, Welch two-sample *t*-tests were used to examine potential baseline differences between groups in continuous demographic characteristics and the number of sessions attended. For these analyses, a significance level of 0.10 was used to ensure that there was no influence of baseline characteristics on the main findings. Additionally, Fisher–Freeman–Halton exact tests were used to examine potential differences between groups in categorical demographic data. Pre-intervention (i.e., baseline) performance data were not tested for potential between-group differences because the statistical model (described below) accounts for any differences. In instances where groups were significantly different in demographic characteristics, Pearson’s correlations, for normally distributed data, and Spearman rho correlations, for non-normally distributed data, were used to examine associations between significantly different demographic variables with baseline balance performance and with task-dependent neural activity to select variables to be included as covariates in the subsequent statistical model. Again, for these analyses, a significance level of 0.10 was used.

To address specific hypotheses related to balance performance, a mixed-effects model analysis was used for each balance condition, controlling for age and time since first brain injury. A random effect for participant was included to account for repeated measures over time. Examination of time effects (fixed effect) were used to determine differences in balance performance (COP_Length_, AP_Range_, and ML_Range_) from pre- to post-intervention time points. The time by group (fixed effect) interaction and linear contrasts were used to examine group differences in changes over time in balance performance. An examination of simple effects was used to determine if there were differences in balance performance from pre- to post-intervention time points within each group.

To address specific hypotheses related to task-dependent neural activity, per fNIRS channel (n = 45) and balance condition, a mixed-effects model, controlling for age and time since first brain injury, was used. A random effect for participant was included to account for repeated measures over time. Examination of time effects (fixed effect) was used to determine differences in task-dependent neural activity (HbO and HbR) from pre- to post-intervention time points. The time by group (fixed effect) interaction and linear contrasts were used to examine group differences in task-dependent neural activity changes over time. Examination of simple effects was used to determine if there were differences in task-dependent neural activity from pre- to post-intervention time points within each group.

For all analyses, adjustments for simple effect comparisons were made using Bonferroni corrections. Results from mixed-effects models are expressed as estimated mean and standard error of the change from pre-intervention to post-intervention (post − pre). Task-dependent neural activity findings from each channel are reported based on the 10-10 standard EEG system and their associated brain area was defined using the Brodmann Atlas from fNIRS Optodes’ Location Decider [34] and depicted using the Brain Function Mapping Tool [48]. Additionally, for all balance performance measurements where significant task-dependent neural activity was observed, Cohen’s d effect size (*d*_Cohen_) was calculated for each time effect and within-group effect (using mean differences and pooled standard deviations) [49]. The following thresholds to interpret Cohen’s d effect sizes were used: 0.20 for small, 0.50 for moderate, 0.80 for large, and 1.30 for very large [50].

## 3. Results

During pre-intervention visits, one male and one female participant were unable to complete the balance conditions due to physical impairments limiting their ability to complete each balance condition, so data were not acquired from them at pre- or post-intervention visits. One male and three female participants did not attend at least seven sessions during the intervention; thus, their data were not included in analyses. Additionally, fNIRS data in one channel (C4–FC4) from one participant were excluded after their anatomical brain scan revealed a lesion in that region. Finally, some channel data were excluded after fNIRS data quality checks. In total, the included data are from 8 men and 15 women that were randomly allocated to either the yoga group (n = 13) or exercise group (n = 10); see Table 1 for demographic characteristics and attendance of each group.

### 3.1. Baseline Differences between Groups

Notably, the average age of the yoga group (mean = 59.1 and SD = 15.3 yrs.) was significantly higher than the average age of the exercise group (mean = 41.0 and SD = 20.8 yrs.) (t(15.99) = 2.32 and *p* = 0.034). Additionally, the average time since first brain injury of the yoga group (mean = 20.2 and SD = 20.9 yrs.) was significantly larger than that of the exercise group (mean = 5.6 and SD = 5.6 yrs.) (t(14.21) = 2.42, *p* = 0.03). There were no significant differences between groups in any other demographic characteristics or the average number of sessions attended (*p*-value range = 0.13–1; see Table 1). Correlation analyses revealed a significant positive relationship between age and pre-intervention balance performance for COP_Length_ for eyes open firm surface (r = 0.37; *p* = 0.079), eyes closed firm surface (r = 0.45; *p* = 0.029), eyes open soft surface (r = 0.51; *p* = 0.015), and eyes closed soft surface (r = 0.47; *p* = 0.029). Additionally, there was a significant positive relationship between time since first brain injury and pre-intervention balance performance for COP_Length_ for eyes open firm surface (r = 0.39; *p* = 0.067), eyes closed firm surface (r = 0.39; *p* = 0.064), and eyes closed soft surface (r = 0.40; *p* = 0.068). As such, age and time since first brain injury were included in subsequent mixed-effects statistical models.

Additionally, correlation analyses revealed numerous significant relationships (both positive and negative) between age and HbO pre-intervention task-dependent neural activity in at least some fNIRS channels for all four balance conditions (*p*-values range = 0.017–0.096). Further, there were numerous significant negative relationships between time since first brain injury and HbO pre-intervention task-dependent neural activity in at least some fNIRS channels for all four balance conditions (*p*-value range = 0.004–0.100). There were also numerous significant relationships (both positive and negative) between age and HbR pre-intervention task-dependent neural activity in at least some fNIRS channels for all four balance conditions (*p*-value range = 0.004–0.086). Lastly, there were significant positive and negative relationships between time since first brain injury and HbR pre-intervention task-dependent neural activity in at least some fNIRS channels for all four balance conditions (*p*-value range = 0.010–0.099). As such, age and time since first brain injury were included in subsequent mixed-effects statistical models.

### 3.2. Balance—Center of Pressure

#### 3.2.1. Balance—Main Effects of Time

**Figure 3:**
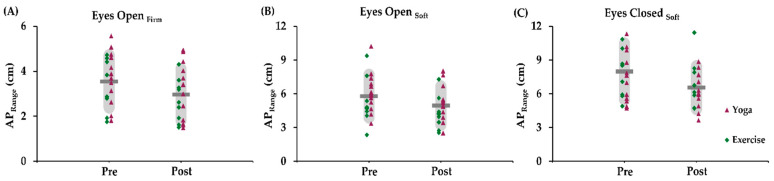
Post-intervention balance improvements. This figure illustrates significant changes in balance performance in the anterior-posterior plane after both interventions. Decreased anterior-posterior displacement (less anterior-posterior instability) represents better balance during (**A**) eyes open firm surface (*p* = 0.005), (**B**) eyes open soft surface (*p* = 0.003), and (**C**) eyes closed soft surface (*p* = 0.011) conditions.

Examination of the mixed-effects model from all included participants, which controlled for age and time since brain injury, indicated that AP_Range_ significantly decreased from pre- to post-intervention in the eyes open firm surface condition (F(1,19.35) = 10.15; *p* = 0.005; Δ(post − pre) = −0.60 ± 0.19 cm; and *d*_Cohen_ = 0.50), eyes open soft surface condition (F(1,18.82) = 11.28; *p* = 0.003; Δ = −0.72 ± 0.21 cm; *d*_Cohen_ = 0.38) and eyes closed soft surface (F(1,16.16) = 8.23; *p* = 0.011; Δ = −0.98 ± 0.34 cm; and *d*_Cohen_ = 0.42) condition (Figure 3). This decrease indicates less anterior-posterior sway—and thus better balance—after the intervention. Additionally, there were significant decreases from pre- to post-intervention for eyes closed soft surface in COP_Length_ (F(1,16.74) = 12.01; *p* = 0.003; Δ = −23.38 ± 6.74 cm; and *d*_Cohen_ = 0.40) and in the ML_Range_ (F(1,16.43) = 21.91; *p* < 0.001; Δ = −1.49 ± 0.32 cm; and *d*_Cohen_ = 0.58). Again, this decrease indicates less sway from the center of pressure and less medial-lateral sway (i.e., better balance) after the intervention.

There were no significant changes from pre- to post-intervention for AP_Range_ in the eyes closed firm surface condition (F(1,19.02) = 2.48; *p* = 0.132; Δ = −0.403 ± 0.256 cm; and *d*_Cohen_ = 0.21). Additionally, there were no significant changes in COP_Length_ in the eyes open firm surface, eyes closed firm surface, and eyes open soft surface conditions (*p*-value range = 0.164–0.193; Δ range = −6.24 ± 4.63 to −5.23 ± 3.68 cm; and *d*_Cohen_ range = 0.12–0.20). Lastly, there were no significant changes in ML_Range_ in the eyes open firm surface, eyes closed firm surface, and eyes open soft surface conditions (*p*-value range = 0.093–0.280; Δ range = −0.26 ± 0.23 to −0.35 ± 0.24 cm; and *d*_Cohen_ range = 0.12–0.23).

#### 3.2.2. Balance—Interaction Effects and Linear Contrasts

Examination of the mixed-effects model, which controlled for age and time since brain injury, indicated that there were no significant group by time interaction effects in COP_Length_ (*p*-value range = 0.365–0.642), ML_Range_ (*p*-value range = 0.187–0.888), and AP_Range_ (*p*-value range = 0.572–0.961) for any balance condition. Furthermore, the linear contrasts revealed that the average change from pre- to post-intervention between the yoga group and the exercise group were not different in COP_Length_ (*p*-value range = 0.365–0.642), ML_Range_ (*p*-value range = 0.187–0.888), and AP_Range_ (*p*-value range = 0.572–0.961) for any balance condition.

#### 3.2.3. Balance—Simple (Within-Group) Effects

In the yoga group, examination of simple effects from the mixed-effects model, which controlled for age and time since brain injury, indicated that AP_Range_ significantly decreased from pre- to post-intervention during the eyes open firm surface condition (F(1,19.54) = 6.19; *p* = 0.022; Δ = −0.61 ± 0.24 cm; and *d*_Cohen_ = 0.49) and the eyes open soft surface condition (F(1,18.56) = 7.57; *p* = 0.013; Δ = −0.73 ± 0.26 cm; and *d*_Cohen_ = 0.36) (Table 2). Additionally, in the exercise group, COP_Length_ significantly decreased from pre- to post-intervention during the eyes closed soft surface condition (F(1,16.82) = 7.70; *p* = 0.013; Δ = −29.24 ± 10.53 cm; and *d*_Cohen_ = 0.47). In both groups, there was a significant decrease from pre- to post-intervention in ML_Range_ during the eyes closed soft surface condition (*p*-values 0.004–0.005; *d*_Cohens_ = 0.50–0.60). Again, these decreases indicate less sway during balance conditions, which represents better balance performance.

### 3.3. Task-Dependent Neural Activity

#### 3.3.1. Task-Dependent Neural Activity—Main Effects of Time

**Figure 4:**
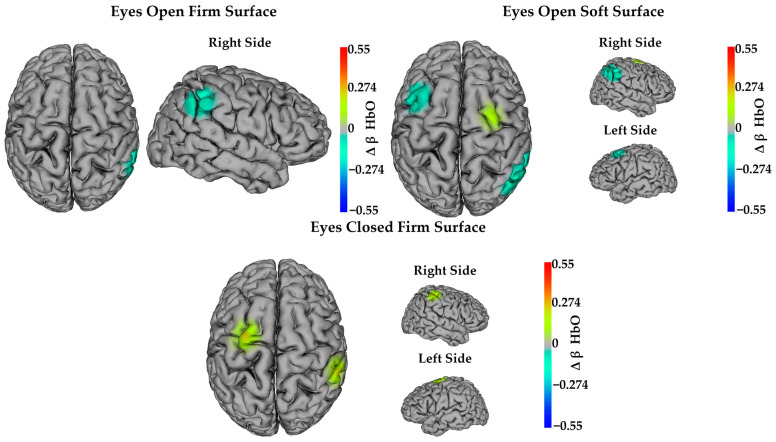
Task-dependent neural activity changes in HbO after both interventions. Heat maps show significant main effects of time (*p* ≤ 0.05). Warm colors (red and yellow) indicate significantly increased activity after both interventions. Cool colors (blue and cyan) indicate significantly decreased activity after both interventions.

Examination of the mixed-effects model, which controlled for age and time since brain injury, indicated that HbO decreased from pre- to post-intervention in both groups in channel CP6–CP4 during the eyes open firm surface condition (Δ = −0.207 ± 0.096; F(1,18.86) = 4.64; *p* = 0.044; and *d_Cohen_* = 0.53) and the eyes open soft surface condition (Δ = −0.183 ± 0.079; F(1,21.09) = 5.33; *p* = 0.031; and *d_Cohen_* = 0.58). Decreased HbO was also observed in both groups in channels FC1–FC3 (Δ = −0.237 ± 0.086; F(1,20.82) = 7.58; *p* = 0.012; and *d_Cohen_* = 0.66) and P4–CP4 (Δ = −0.216 ± 0.091; F(1,20.78) = 5.62; *p* = 0.028; and *d_Cohen_* = 0.64) during the eyes open soft surface condition. In contrast, HbO increased from pre- to post-intervention in both groups in channels FC1–C1 (Δ = 0.281 ± 0.074; F(1,20.91) = 14.57; *p* = 0.001; and *d_Cohen_* = 0.91) and C4–CP4 (Δ = 0.262 ± 0.092; F(1,18.74) = 8.20; *p* = 0.01; and *d_Cohen_* = 0.71) during the eyes closed firm surface condition. HbO also increased in both groups in channel FC2–C2 (Δ = 0.173 ± 0.079; F(1,19.95) = 4.78; *p* = 0.041; and *d_Cohen_* = 0.76) during the eyes open soft surface condition. Figure 4 illustrates significant changes in task-dependent neural activity from pre- to post-intervention.

In HbR, examination of the mixed-effects model indicated there was a decrease from pre- to post-intervention in both groups in channel P4–CP4 during the eyes open firm surface condition (Δ = −0.329 ± 0.109; F(1,20.72) = 9.12; *p* = 0.007; and *d*_Cohen_ = 0.90). Furthermore, HbR increased from pre- to post-intervention in channel C3–C5 (Δ = 0.194 ± 0.080; F(1,17.78) = 5.85; *p* = 0.027; and *d*_Cohen_ = 0.46) during the eyes open soft surface condition.

#### 3.3.2. Task-Dependent Neural Activity—Interaction Effects and Linear Contrasts

The mixed-effects model, which controlled for age and time since brain injury, revealed significant group by time HbO interaction effects. These effects were present in channels C4–C6 and CP2–CP4 during the eyes open soft surface (*p*-values = 0.034 and 0.026, respectively) and eyes closed soft surface (*p*-values = 0.022 and 0.017, respectively) conditions. Specifically, in the yoga group, HbO decreased during both balance conditions from pre- to post-intervention in channels C4–C6 and CP2–CP4. In the exercise group, HbO increased during both conditions from pre- to post-intervention in channel C4–C6 and CP2–CP4. Interaction effects were also present in channels P4–P6 (F(1,21.01) = 4.62; *p* = 0.043) and P4–P2 (F(1,21.40) = 5.87; *p* = 0.024) for eyes open firm surface. Specifically, in the yoga group, HbO increased with time in channels P4–P6 and P4–P2. In the exercise group, HbO decreased with time in channels P4–P6 and P4–P2. Additionally, interaction effects in HbO were present in channels T7–FT7 (F(1,19.56) = 4.42; *p* = 0.049) and FC5–FC3 (F(1,18.13) = 4.69; *p* = 0.044) for the eyes open soft surface condition. Specifically, in the yoga group, HbO increased with time in channel T7–FT7 and decreased in channel FC5–FC3. In the exercise group, HbO decreased in channel T7–FT7 and increased in channel FC5–FC3. Lastly, interaction effects in HbO were present in channels T7–C5, CP5–C5, CP5–CP3, P3–CP3, CP6–CP4, C4–CP4, and C4–C2 (*p*-value range = 0.003–0.043) for eyes closed soft surface. Specifically, in the yoga group, HbO increased with time in channel T7–C5, and in the exercise group decreased with time. In contrast, in the yoga group, HbO decreased with time in channels CP5–C5, CP5–CP3, P3–CP3, CP6–CP4, and C4–C2, while in the exercise group, it increased with time in the same channels.

The linear contrast confirmed that significant interaction effects were present, as described above. Specifically, the linear contrast revealed that the estimated difference in change in HbO from pre- to post-intervention between both groups was significant. The estimated difference in change over time was significant in channels C4–C6 and CP2–CP4 during the eyes open soft surface (*p*-values = 0.034 and 0.026, respectively) and eyes closed soft surface (*p*-values = 0.022 and 0.017, respectively) conditions (Table 3). The estimated difference was also significant in channels P4–P6 (*p* = 0.043) and P4–P2 (*p* = 0.024) during the eyes open firm surface condition. Additionally, estimated differences were significant in channels T7–FT7 (*p* = 0.049) and FC5–FC3 (*p* = 0.044) for eyes open soft surface. Lastly, estimated differences in change were significant in channels T7–C5, CP5–C5, CP5–CP3, P3–CP3, CP6–CP4, C4–CP4, and C4–C2 (*p*-value range = 0.003–0.043) for eyes closed soft surface.

In HbR, the mixed-effects model, which controlled for age and time since brain injury, also revealed significant group by time interaction effects. These were present in channel CP5–P5 during the eyes open firm surface (F = 5.79(1,16.42); *p* = 0.028), eyes open soft surface (F(1,16.59) = 7.40; *p* = 0.015), and eyes closed soft surface (F(1,17.65) = 4.46; *p* = 0.049) conditions. Specifically, in the yoga group, HbR decreased with time during the three conditions. In the exercise group, HbR increased with time during the three conditions. Significant interaction effects in HbR were also present in channel Cz–C2 for all balance (*p*-value range = 0.002–0.026) conditions. Specifically, in the yoga group, HbR decreased with time during all conditions. In the exercise group, HbR increased with time during all conditions. Additional interaction effects in HbR were present in channel T8–C6 during the eyes open firm surface (F(1,17.26) = 5.89; *p* = 0.026) and eyes open soft surface (F(1,20.34) = 7.80; *p* = 0.011) conditions. Specifically, in the yoga group, HbR decreased with time during both conditions. In the exercise group, HbR increased with time during both conditions. Further interaction effects were present in channel CP6–CP4 for eyes open soft surface (F(1,20.46) = 5.26; *p* = 0.033) and eyes closed firm surface (F(1,20.72) = 6.53; *p* = 0.019) conditions. Specifically, in the yoga group, HbR decreased with time during both conditions. In the exercise group, HbR increased with time during both conditions. Similarly, interaction effects were present in channels FC2–FC4 (F(1,21.02) = 4.48; *p* = 0.046) and FC2–C2 (F(1,21.01) = 6.97; *p* = 0.015) during the eyes open firm surface condition. Again, in the yoga group, HbR decreased with time in both channels, but in the exercise group, HbR increased with time. Finally, significant interaction effects were observed in channels CP5–CP3, FC5–FC3, FC6–FT8, P4–CP4, and C4–C6 in HbR during the eyes open soft surface (*p*-values range = 0.015–0.047) condition. Specifically, in the yoga group, HbR increased with time in channel CP5–CP3, while it decreased with time in all the other channels. Conversely, in the exercise group, HbR increased with time in all channels.

The linear contrast confirmed that significant interaction effects were present, as described above. Specifically, the linear contrast revealed that the estimated difference in change in HbR from pre- to post-intervention between groups was significant. The estimated difference in change over time was significant in channel CP5–P5 during the eyes open firm surface (*p* = 0.028), eyes open soft surface (*p* = 0.015), and eyes closed soft surface (*p* = 0.049) conditions. The estimated difference was also significant in channel Cz–C2 during all balance (*p*-value range = 0.002–0.026) conditions. The estimated difference was significant in channel T8–C6 during the eyes open firm surface (*p* = 0.026) and eyes open soft surface (*p* = 0.011) conditions. Additionally, the estimated difference was significant in channel CP6–CP4 during the eyes open soft surface (*p* = 0.033) and eyes closed firm surface (*p* = 0.019) conditions. The estimated difference was also significant in channels FC2–FC4 (*p* = 0.046) and FC2–C2 (*p* = 0.015) during the eyes open firm surface condition. Lastly, the estimated difference was significant in channels CP5–CP3, FC5–FC3, FC6–FT8, P4–CP4, and C4–C6 during the eyes open soft surface (*p*-values range = 0.015–0.047) condition (Table 4).

#### 3.3.3. Task-Dependent Activity—Simple (Within-Group) Effects

In the yoga group, examination of simple effects from the mixed-effects model, which controlled for age and time since brain injury, indicated that HbO decreased from pre- to post-intervention in channels FC5–FC3, FC1–FC3, and CP2–CP4 during the eyes open soft surface (*p*-value range = 0.005–0.019) condition. Also, in the yoga group, HbO decreased in channel P3–CP3 during the eyes closed soft surface (Δ = −0.236 ± 0.087; F(1,20.55) = 7.39; *p* = 0.013) condition. In contrast, in the yoga group, HbO increased from pre- to post-intervention in channel FC1–C1 during the eyes closed firm surface (Δ = 0.334 ± 0.095; F(1,20.37) = 13.02; *p* = 0.002) condition.

**Figure 5:**
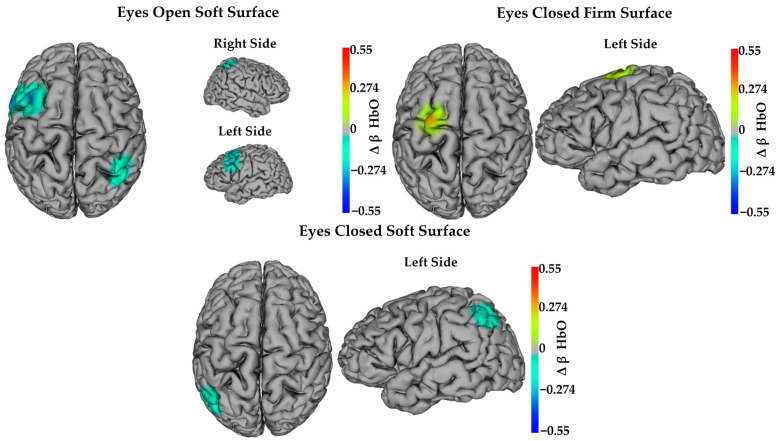
Task-dependent neural activity changes in HbO within the yoga group. Heat maps illustrate significant simple (within-group) effects of time after Bonferroni correction (*p* ≤ 0.025). Warm colors (red and yellow) indicate significantly increased activity after both interventions. Cool colors (blue and cyan) indicate significantly decreased activity after both interventions.

**Figure 6:**
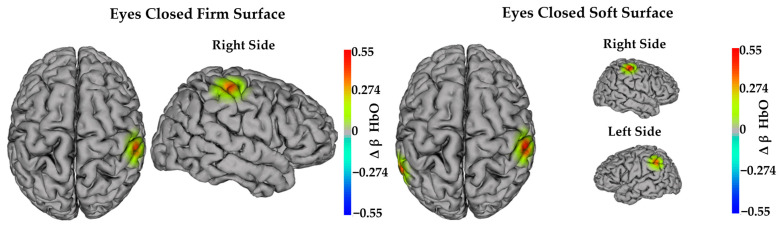
Task-dependent neural activity changes in HbO within the exercise group. Heat maps illustrate significant simple (within-group) effects of time after Bonferroni correction (*p* ≤ 0.025). Warm colors (red and yellow) indicate significantly increased activity after both interventions. Cold colors (blue and cyan) indicate significantly decreased activity after both interventions.

For the exercise group, HbO increased in channel C4–CP4 during eyes closed firm surface and eyes closed soft surface (*p*-values = 0.008 and 0.005, respectively) conditions. Also, in the exercise group, HbO increased in channel CP5–CP3 during the eyes closed soft surface (Δ = 0.355 ± 0.102; F(1,21.05) = 12.08; and *p* = 0.002) condition. Figure 5 and Figure 6 illustrate significant simple (within-group) effects in task-dependent neural activity per group. Specific details of significant simple (within-group) effects in task-dependent neural activity per group are provided in Table 5.

In the yoga group, examination of simple effects from the mixed-effects model indicated that HbR decreased from pre- to post-intervention in channel Cz–C2 during all balance (*p*-value range = 0.003–0.024) conditions. Also, in the yoga group, HbR decreased in channel P4–CP4 during the eyes open firm surface (Δ = −0.493 ± 0.141; F(1,20.09) = 12.14; and *p* = 0.002), eyes open soft surface (Δ = −0.412 ± 0.140; F(1,20.08) = 8.65; and *p* = 0.008), and eyes closed firm surface (Δ = −0.407 ± 0.139; F(1,20.69) = 8.51; and *p* = 0.008) conditions. Moreover, in the yoga group, HbR decreased in channels CP6–CP4 and C4–FC4 during the eyes closed firm surface (*p*-values = 0.022 and 0.016, respectively) condition. Lastly, in the yoga group, HbR decreased in channel FC2–FC4 during the eyes open firm surface (Δ = −0.309 ± 0.123; F(1,20.50) = 6.31; and *p* = 0.020) condition.

In the exercise group, HbR increased from pre- to post-intervention in channels CP5–CP3 and T8–C6 during the eyes open soft surface (*p*-values = 0.007 and 0.015, respectively) condition (Table 6).

## 4. Discussion

The purpose of this study was to compare the impact of eight weeks of yoga and exercise on balance and task-dependent neural activity in adults with chronic ABI. We found that, regardless of intervention, participants had significant improvements in balance performance from pre- to post-intervention. Specifically, we observed that after the intervention, participants had significantly reduced postural instability, primarily in the anterior to posterior plane, during three of four balance conditions. In general, this finding aligns with previous research—including our own—showing that yoga and other forms of exercise can improve balance in individuals with ABI [10,12]. Notably, in many prior studies, balance performance has been measured with clinical assessments, like the Berg Balance Scale, which has been validated for individuals with ABI [51]. In contrast, we used a force plate to assess balance performance, and we remained able to detect that yoga—along with a comparable, control exercise intervention—improved balance performance in adults with ABI. Importantly, the sensor technology within the force plate allowed us to detect specific reductions in anterior to posterior sway, while general reductions in sway and reduced medial to lateral sway were also detected in the most challenging balance condition—eyes closed on a soft/unstable surface. Adults with ABI, specifically stroke, are known to have greater sway in the anterior to posterior plane [52], so there may have been greater room for improvement in this aspect of postural stability. However, the force plate was also able to detect other reductions in sway during a more difficult condition (i.e., eyes closed on a soft/unstable surface), suggesting that the force plate might be a particularly useful tool to detect discrete aspects of balance performance in clinical populations.

Notably, there were no significant differences in the degree of balance improvement between the yoga group and the low-impact exercise group. The absence of between-group differences might be explained by the similar intensity used for both interventions, which was intentional. It is also possible that simply participating in a community-based group activity was beneficial to adults with ABI. Our previous work has consistently shown that eight weeks of yoga improves balance in adults with ABI [11,12,13]. However, until this study, it was unclear if yoga had unique characteristics that supported balance improvement or if group activity would support balance improvement. It is well established that adults with ABI frequently have adynamia, or reduced motivation, which can make it difficult to self-initiate activities [53], like exercise. Both of our interventions provided structured activities and external motivation for individuals with ABI. Further, these interventions required that participants leave their homes, drive or find transportation at a set time twice a week, and engage with others in a community setting. These activities alone, independent of the physical exercise completed in the classes, could have challenged their balance in ways that supported improvement. Alternatively, the intentional similarity of the yoga and exercise classes may have similarly supported improvements in balance. In future work, additional control groups (e.g., a group activity or education with no exercise component) could further elucidate what contributes to improved balance in adults with ABI who engage in group activity. Nonetheless, our preliminary findings suggest that either yoga or exercise can improve balance in ABI. Importantly, this finding provides evidence-based options for people with ABI and their clinical providers when they are seeking to improve balance.

In addition to examining balance performance after the two interventions, we also examined changes in task-dependent neural activity after yoga or exercise. Specifically, we examined brain activity during balance conditions, which allowed us to examine the neural underpinnings of improved balance performance. We found that, regardless of intervention, task-dependent neural activity was decreased post-intervention in the right supramarginal gyrus during the eyes open balance conditions. It also decreased in the left premotor cortex and in the right angular gyrus during the eyes open soft surface condition. We also found that task-dependent neural activity was increased post-intervention in the left supplementary motor area and right supramarginal gyrus during the eyes closed firm surface condition. Neural activity was also increased in the right supplementary motor area during the eyes open soft surface condition. To interpret these findings, we considered the role of each of these brain regions, where significant increases and decreases were observed.

The supramarginal gyrus (Brodmann area 40) and the angular gyrus (Brodmann area 39) are linked to spatial discrimination and visuomotor function [54], respectively. Both gyri are part of the inferior parietal lobule [55], which is thought to be involved in sensorimotor integration [56]. Additionally, the function of the premotor cortex and supplementary motor area (both part of the Brodmann area 6) is motor planning. Specifically, the premotor cortex is involved in motor planning that uses learned associations between cues and responses [57]. The supplementary motor area supports voluntary movement and uses internal attention to engagement in movement, as opposed to responding to external cues [58]. Taken together, it appears that, regardless of intervention group, participants with chronic ABI had higher neural activity in regions that automatically respond to the balance task demands and lower neural activity in regions that respond to external cues.

This explanation should be interpreted cautiously due to the novelty of our study and the paucity of intervention studies to which we could compare our findings. In cross-sectional studies that have used fNIRS in healthy populations [55,59] and in stroke [25], the supramarginal gyrus [55,59], premotor cortex [25,55], and supplementary motor area [25,55] have had increased task-related activity during different balance conditions. Additionally, although the angular gyrus has been examined with fNIRS during balance [55], there has not been sufficient evidence of its involvement during different balance conditions. However, an fMRI study that asked participants to imagine standing found that the angular gyrus was active during such a task [60]. Still, it is unclear if the angular gyrus activity supports balance performance, and further studies using neuroimaging methods that support free movement, like fNIRS, are needed.

In sum, our current results show significant changes in neural activity in brain regions that do not fully align with previous work. However, due to significant methodological differences (e.g., cross-sectional vs. RCT), it is difficult to directly compare findings. To our knowledge, there is only one study comparing the time effects of a combined intervention of physical activity and occupational therapy on participants with stroke [27]. The authors found that after the intervention, there was increased activity in bilateral supplementary motor area activity during backward–forward balance perturbations [27]. This finding aligns with our finding of increased neural activity in the supplementary motor area during balance, which is encouraging. Still, much more research is needed to understand the neural underpinnings of balance improvements in adults with ABI. Further use of novel neuroimaging methods like portable fNIRS may allow us to gain additional understanding.

Although there was not sufficient evidence of significant differences between groups in balance performance, we examined if there were any differences between groups in change over time (i.e., from pre- to post-intervention) in neural activity. In other words, we sought to understand if the change over time in the yoga group was different from the change over time in the exercise group. For that purpose, we tested for interaction effects and confirmed findings with linear contrasts. We found significant differences from pre- to post-intervention between groups in task-dependent neural activity in multiple brain regions. During the eyes open firm surface condition, there was a significant difference between groups in the right angular gyrus and right somatosensory association cortex (Brodmann area 7). Specifically, HbO increased from pre- to post-intervention in the yoga group, but it decreased from pre- to post-intervention in the exercise group. Additionally, during the eyes open soft surface condition, there was a significant difference between groups in the left middle temporal gyrus (Brodmann area 21), left pars opercularis (Brodmann area 44), right somatosensory cortex (Brodmann area 1–3), and right supramarginal gyrus. Specifically, HbO increased from pre- to post-intervention in the left middle temporal gyrus, but it decreased from pre- to post-intervention in the other brain regions in the yoga group. Conversely, in the exercise group, HbO decreased from pre- to post-intervention in the left middle temporal gyrus, but it increased from pre- to post-intervention in the other brain regions. Lastly, for the eyes closed soft surface condition, there was a significant difference between groups in the left superior temporal gyrus (Brodmann area 22), left retrosubicular area (Brodmann area 48), bilateral supramarginal gyrus, left angular gyrus, right somatosensory cortex, and right primary motor cortex (Brodmann area 4). Specifically, HbO increased from pre- to post-intervention in the left superior temporal gyrus, but it decreased from pre- to post-intervention in the other brain regions in the yoga group. Conversely, in the exercise group, HbO decreased from pre- to post-intervention in the left superior temporal gyrus, but it increased from pre- to post-intervention in the other brain regions.

To fully understand these findings, we carefully examined the changes from pre- to post-intervention within the yoga group and the exercise group. Within the yoga group, we found that task-dependent neural activity decreased in the left pars opercularis, left premotor cortex, and right supramarginal gyrus during the eyes open soft surface condition. Also, the task-dependent neural activity decreased in the left angular gyrus during the eyes closed soft surface condition. In contrast, task-dependent neural activity increased in the left supplementary motor area during the eyes closed firm surface condition. Again, we considered the role of each of these brain regions to interpret our findings. The pars opercularis is part of the inferior frontal gyrus [61], and Takakura and colleagues [55] reported increased task-dependent neural activity of the right frontal operculum/inferior frontal gyrus during complex balance tasks. Unfortunately, the authors did not measure task-dependent neural activity from left pars opercularis, so it is unclear if similar activity would be observed in the left hemisphere. Nevertheless, the left and right inferior frontal gyrus, mainly the pars opercularis, respond to vestibular stimulation [62]. As such, our findings from the yoga group may indicate that participants had less reliance on neural resources associated with vestibular system function during balance tasks.

Notably, many of the within-group findings in the yoga group align with the overall study results (i.e., those showing a main effect of time from pre- to post-intervention). Specifically, participants in the yoga group had less neural activity in brain regions linked to spatial discrimination (supramarginal gyrus), visuomotor function (angular gyrus), and motor planning from external cues (premotor cortex). They also had more neural activity in regions associated with motor planning from internal cues (supplemental motor area). In sum, it appears that many of the overall study results from pre- to post-intervention were driven by the yoga group. Thus, there may be unique features of yoga that elicited these specific changes in adults with ABI. Yoga emphasizes mindfulness and a mind–body connection through breathing and meditation techniques. According to Rivest-Gadbois and Boudrias [17], yoga may increase body awareness, interoception, and attentional control. It is, therefore, possible that the decreased activity in the premotor cortex and the increased activity in the supplementary motor area reflected improvements in interoception. Nevertheless, this explanation should be interpreted cautiously, as much more research is needed to understand how yoga, or other interventions, influences the neural underpinnings of balance improvements.

Finally, we examined the unique changes in task-dependent neural activity in the exercise group. There was increased neural activity in the right supramarginal gyrus during eyes closed firm surface and eyes closed soft surface conditions. There was also increased neural activity in the left supramarginal gyrus during the eyes closed soft surface condition. In contrast to the yoga group, where the activity of the supramarginal gyrus decreased post-intervention, participants in the exercise group had increased activity over the supramarginal gyrus post-intervention. Overall, it appears that the balance improvements experienced by participants in the exercise group were elicited by different brain changes than those seen in the yoga group. Specifically, it appears that the exercise participants improved balance by allocating more neural resources to regions of the brain that support spatial discrimination and sensorimotor integration. Thus, there may have been unique features of both interventions that elicited changes in different neural underpinnings to support balance improvement. However, this potential explanation should also be interpreted cautiously as significantly more research is needed to confirm these types of conclusions.

### Limitations and Future Directions

Although significant effort and resources were allocated to recruit participants through various methods (for details see Stephens et al. [29]), our sample size was quite small and heterogenous (e.g., age range of the sample was 19–82 years; the time since first brain injury ranged from 0.84 to 62 years). The small heterogenous sample may have limited our ability to detect significant changes. Also, our study consisted of 23 white, non-Hispanic participants, which is not fully representative of the local community nor the brain injury population at large. This, and other characteristics of our sample, likely influence the generalizability of our findings. Still, it is promising that significant improvements in balance were observed, along with significant changes in task-dependent neural activity in this pilot sample. As mentioned throughout the Discussion, significantly more research is needed to elucidate the neural underpinnings of balance in clinical populations, like ABI, and to elucidate how neural structures change to support balance after an intervention.

There were also some methodological challenges that may have influenced our findings. For example, some (but not all) participants experienced fatigue during the pre- and/or post-intervention assessments and required resting time between balance trials. Participants were given time to sit and rest between balance trials, and this change in position from sitting to standing may have influenced blood pressure and cerebral perfusion, but it was necessary to maintain safety. It is also possible that the participants who needed to sit were experiencing fatigue that could have altered their balance performance. For safety purposes, it was essential that participants be allowed to rest, but this could have meaningfully changed our fNIRS and balance data. Future studies should quantify changes (if any) in fNIRS and/or balance outcomes based on body position or self-reported fatigue level.

Finally, the relative newness of fNIRS methodology, as compared to other neuroimaging methods (e.g., EEG and fMRI), results in fewer studies that can be used to inform methodology, data analysis steps, and data interpretation. Nonetheless, novel studies like this one are essential for addressing important empirical questions and for providing a foundation for future replicative studies with larger sample sizes and populations from different races or ethnicities.

## 5. Conclusions

Advances in sensor technology, such as the sensors used in force plates and neuroimaging devices like fNIRS, support advances in neuroimaging and neurorehabilitation research. Here, using such technology, we found that yoga and exercise improved balance performance and significantly changed task-dependent neural activity in adults with chronic ABI. These preliminary results provide an important foundation for continued neurorehabilitation work with neuroimaging methods, like portable fNIRS.

## Figures and Tables

**Table 1:** Demographic characteristics of sample.

	Groups		
	Yoga (n = 13)	Exercise (n = 10)	
	Mean (SD)	Mean (SD)	*p*-Value
Age (years)	59.15 ± 15.32	41.00 ± 20.81	0.03 *
Sessions Attended	13.31 ± 2.87	12.70 ± 2.36	0.58
Time Since First Brain Injury (years)	20.23 ± 20.88	5.58 ± 5.64	0.03 *
Sex	n (%)	n (%)	
Male	3 (23)	5 (50)	0.13
Female	10 (77)	5 (50)	
Race/Ethnicity			
White	13 (100)	10 (100)	NA
Educational Level			
High School	0 (00)	3 (30)	0.15
Some College	3 (23)	1 (10)	
College Graduate	6 (46)	2 (20)	
Some Post-Graduate	1 (08)	0 (00)	
Post-Graduate Degree	3 (23)	4 (40)	
ABI Type			
Aneurysm	0 (00)	1 (10)	0.94
Carcinoma	1 (08)	0 (00)	
Hypoxia	1 (08)	1 (10)	
Stroke	4 (31)	3 (30)	
TBI	7 (54)	5 (50)	
Previous Rehabilitation			
Yes	10 (77)	8 (80)	1.00
No	3 (23)	2 (20)	
Self-Reported Loss of Balance			
Moderate	10 (77)	7 (70)	0.81
Moderate to Severe	2 (15)	1 (10)	
Severe	1 (08)	2 (20)	
Depression			
Yes	10 (77)	7 (70)	1.00
No	3 (23)	3 (30)	
ADD/ADHD			
Yes	3 (23)	1 (10)	0.60
No	10 (77)	9 (90)	
Sensory Processing Difficulties			
Yes	2 (15)	3 (30)	0.62
No	11 (85)	7 (70)	
Fainting Episodes?			
Yes	7 (54)	4 (40)	0.68
No	6 (46)	6 (60)	

Note: Summary statistics (e.g., mean, standard deviation (SD), frequency (percentage)) for demographic characteristics (e.g., age and sex) and attendance for participants in both the yoga and the exercise interventions. *p*-values for tests of baseline differences between intervention groups are also included. * denotes significant difference between intervention groups.

**Table 2:** Detailed balance results for each group.

	Yoga				Exercise			
Condition	Est. Mean ± SE Δ (cm)	F(df)	*p*	*d*_Cohen_	Est. Mean ± SE Δ (cm)	F(df)	*p*	*d*_Cohen_
AP_Range_ EO_Firm_	−0.61 ± 0.24	6.19(1,19.54)	0.022 *	0.49	−0.58 ± 0.28	4.25(1,19.21)	0.053	0.46
AP_Range_ EO_Soft_	−0.73 ± 0.26	7.57(1,18.56)	0.013 *	0.36	−0.71 ± 0.33	4.44(1,18.99)	0.049	0.35
AP_Range_ EC_Firm_	−0.55 ± 0.33	2.71(1,19.16)	0.116	0.27	−0.26 ± 0.39	0.44(1,18.92)	0.517	0.12
AP_Range_ EC_Soft_	−1.05 ± 0.43	5.91(1,15.65)	0.028	0.42	−0.92 ± 0.53	2.95(1,16.29)	0.105	0.37
COP_Length_ EO_Firm_	−1.97 ± 4.86	0.16(1,20.18)	0.690	0.07	−8.98 ± 5.82	2.39(1,20.52)	0.138	0.30
COP_Length_ EO_Soft_	−4.06 ± 5.47	0.50(1,19.23)	0.488	0.10	−8.42 ± 7.26	1.34(1,19.71)	0.260	0.20
COP_Length_ EC_Firm_	−8.04 ± 4.71	2.91(1,20.07)	0.103	0.18	−2.42 ± 5.65	0.18(1,20.22)	0.673	0.05
COP_Length_ EC_Soft_	−17.52 ± 8.46	4.29(1,16.41)	0.054	0.28	−29.24 ± 10.53	7.70(1,16.82)	0.013 *	0.47
ML_Range_ EO_Firm_	−0.28 ± 0.23	1.60(1,19.73)	0.221	0.20	−0.33 ± 0.27	1.54(1,20.06)	0.229	0.24
ML_Range_ EO_Soft_	−0.52 ± 0.30	3.02(1,18.55)	0.099	0.22	−0.19 ± 0.38	0.25(1,18.96)	0.622	0.08
ML_Range_ EC_Firm_	−0.58 ± 0.30	3.74(1,19.48)	0.068	0.13	0.06 ± 0.36	0.03(1,19.72)	0.869	0.16
ML_Range_ EC_Soft_	−1.35 ± 0.40	11.45(1,16.06)	0.004 *	0.50	−1.63 ± 0.50	10.73(1,16.52)	0.005 *	0.60

Note: Simple (within-group) effects of time in balance performance per intervention group during the different balance conditions. Estimated mean changes (post − pre), standard errors, F statistic with degrees of freedom, *p*-values, and Cohen’s d are presented. * Denotes significant difference after Bonferroni correction (*p* ≤ 0.025). Abbreviations: SE: standard error; EO_Firm_: eyes open firm surface; EO_Soft_: eyes open soft surface; EC_Firm_: eyes closed firm surface; EC_Soft_: eyes closed soft surface; AP_Range_: anterior-posterior range; COPLength; center of pressure length; and MLRange: medial-lateral range.

**Table 3:** Interaction effects, within-group change, and linear contrast results for HbO.

			Interaction Effects Group Change (Post − Pre)				Linear Contrast		
Channel (10-10)	Landmark (Hemisphere)	Balance Condition	F(df)	*p*	Δ Exercise (Mean ± SE)	Δ Yoga (Mean ± SE)	t(df)	*p*	Estimated Difference ± SE (ΔE − ΔY)
P4–P6	AG (R)	EO_Firm_	4.62(1,21.01)	0.043	−0.170 ± 0.121	0.174 ± 0.105	−2.15(21.01)	0.043	−0.334 ± 0.160
P4–P2	SAC (R)	EO_Firm_	5.87(1,21.40)	0.024	−0.326 ± 0.151	0.157 ± 0.130	−2.42(21.40)	0.024	−0.483 ± 0.199
T7–FT7	MTG (L)	EO_Soft_	4.42(1,19.56)	0.049	−0.199 ± 0.149	0.215 ± 0.129	−2.10(19.56)	0.049	−0.414 ± 0.197
FC5–FC3	POP (L)	EO_Soft_	4.69(1,18.13)	0.044	0.058 ± 0.141	−0.353 ± 0.127	2.17(18.13)	0.044	0.411 ± 0.190
C4–C6	S1 (R)	EO_Soft_	5.13(1,20.56)	0.034	0.111 ± 0.114	−0.226 ± 0.096	2.26(20.56)	0.034	0.338 ± 0.149
CP2–CP4	SMG (R)	EO_Soft_	5.85(1,18.82)	0.026	0.100 ± 0.102	−0.227 ± 0.088	2.42(18.82)	0.026	0.326 ± 0.135
T7–C5	STG (L)	EC_Soft_	6.54(1,20.75)	0.018	−0.247 ± 0.142	0.229 ± 0.120	−2.56(20.75)	0.018	−0.476 ± 0.186
CP5–C5	RA (L)	EC_Soft_	4.76(1,20.86)	0.041	0.179 ± 0.127	−0.182 ± 0.107	2.18(20.86)	0.041	0.362 ± 0.166
CP5–CP3	SMG (L)	EC_Soft_	11.21(1,20.76)	0.003	0.355 ± 0.102	−0.092 ± 0.086	3.35(20.76)	0.003	0.447 ± 0.134
P3–CP3	AG (L)	EC_Soft_	9.19(1,21.09)	0.006	0.172 ± 0.103	−0.236 ± 0.087	3.03(21.09)	0.006	0.408 ± 0.134
CP6–CP4	SMG (R)	EC_Soft_	5.02(1,20.64)	0.036	0.189 ± 0.113	−0.141 ± 0.095	2.24(20.64)	0.036	0.330 ± 0.147
C4–C6	S1 (R)	EC_Soft_	6.14(1,20.45)	0.022	0.243 ± 0.118	−0.139 ± 0.099	2.48(20.45)	0.022	0.382 ± 0.154
C4–CP4	SMG (R)	EC_Soft_	7.40(1,21.06)	0.013	0.405 ± 0.131	−0.060 ± 0.110	2.72(21.06)	0.013	0.465 ± 0.171
C4–C2	M1 (R)	EC_Soft_	4.79(1,17.12)	0.043	0.316 ± 0.170	−0.169 ± 0.142	2.19(17.12)	0.043	0.485 ± 0.222
CP2–CP4	SMG (R)	EC_Soft_	6.72(1,20.89)	0.017	0.272 ± 0.121	−0.137 ± 0.102	2.59(20.89)	0.017	0.409 ± 0.158

Note: This table includes significant interaction effects, estimated mean changes from pre- to post-intervention within each group, and liner contrast results for HbO for different balance conditions in multiple channels. Channels are labeled using 10-10 coordinates, with specific anatomical landmarks and hemispheres (right and left) identified. Abbreviations: AG: Angular Gyrus; SAC: Somatosensory Association Cortex; MTG: Middle Temporal Gyrus; POP: Pars Opercularis; S1: Primary Somatosensory Cortex; SMG: Supramarginal Gyrus; STG: Superior Temporal Gyrus; RA: Retrosubicular Area; M1: Primary Motor Cortex. EO_Firm_: Eyes Open Firm Surface; EO_Soft_: Eyes Open Soft Surface; EC_Soft_: Eyes Closed Soft Surface; R = right hemisphere; L = left hemisphere; Post = post-intervention; Pre = pre-intervention; Δ = change; SE = standard errors; E = exercise Group; Y = yoga Group; and df: degrees of freedom.

**Table 4:** Interaction effects, within-group change, and linear contrast results for HbR.

			Interaction Effects		Group Change (Post − Pre)		Linear Contrast		
Channel (10-10)	Landmark (Hemisphere)	Balance Condition	F(df)	*p*	Δ Exercise (Mean ± SE)	Δ Yoga (Mean ± SE)	t(df)	*p*	Estimated Difference ± SE (ΔE − ΔY)
CP5–P5	AG (L)	EO_Firm_	5.79(1,16.42)	0.028	0.240 ± 0.134	−0.174 ± 0.109	2.41(16.42)	0.028	0.415 ± 0.172
Cz–C2	SMA (R)	EO_Firm_	5.74(1,20.36)	0.026	0.225 ± 0.211	−0.436 ± 0.179	2.40(20.36)	0.026	0.662 ± 0.276
T8–C6	STG (R)	EO_Firm_	5.89(1,17.26)	0.026	0.196 ± 0.157	−0.291 ± 0.126	2.43(17.26)	0.026	0.488 ± 0.201
FC2–FC4	PMC (R)	EO_Firm_	4.48(1,21.02)	0.046	0.094 ± 0.145	−0.309 ± 0.123	2.12(21.02)	0.046	0.403 ± 0.190
FC2–C2	SMA (R)	EO_Firm_	6.97(1,21.01)	0.015	0.332 ± 0.177	−0.282 ± 0.151	2.64(21.01)	0.015	0.614 ± 0.232
CP5–P5	AG (L)	EO_Soft_	7.40(1,16.59)	0.015	0.340 ± 0.160	−0.223 ± 0.131	2.72(16.59)	0.015	0.563 ± 0.207
CP5–CP3	SMG (L)	EO_Soft_	5.13(1,18.11)	0.036	0.418 ± 0.137	0.023 ± 0.108	2.26(18.11)	0.036	0.395 ± 0.174
FC5–FC3	POP (L)	EO_Soft_	6.96(1,20.70)	0.015	0.355 ± 0.184	−0.283 ± 0.156	2.64(20.70)	0.015	0.638 ± 0.242
Cz–C2	SMA (R)	EO_Soft_	9.60(1,20.85)	0.005	0.287 ± 0.210	−0.566 ± 0.178	3.10(20.85)	0.005	0.854 ± 0.276
T8–C6	STG (R)	EO_Soft_	7.80(1,20.34)	0.011	0.368 ± 0.139	−0.138 ± 0.117	2.79(20.34)	0.011	0.507 ± 0.181
FC6–FT8	RA (R)	EO_Soft_	6.28(1,20.81)	0.021	0.336 ± 0.151	−0.158 ± 0.127	2.51(20.81)	0.021	0.494 ± 0.197
CP6–CP4	SMG (R)	EO_Soft_	5.26(1,20.46)	0.033	0.277 ± 0.156	−0.194 ± 0.133	2.29(20.46)	0.033	0.471 ± 0.206
P4–CP4	AG (R)	EO_Soft_	6.84(1,20.71)	0.016	0.153 ± 0.164	−0.412 ± 0.140	2.62(20.71)	0.016	0.565 ± 0.216
C4–C6	S1 (R)	EO_Soft_	4.59(1,17.16)	0.047	0.128 ± 0.126	−0.241 ± 0.117	2.14(17.16)	0.047	0.368 ± 0.172
Cz–C2	SMA (R)	EC_Firm_	12.06(1,19.52)	0.002	0.272 ± 0.161	−0.461 ± 0.136	3.47(17.52)	0.002	0.733 ± 0.211
CP6–CP4	SMG (R)	EC_Firm_	6.53(1,20.72)	0.019	0.185 ± 0.148	−0.309 ± 0.125	2.56(20.72)	0.019	0.494 ± 0.193
CP5–P5	AG (L)	EC_Soft_	4.46(1,17.65)	0.049	0.192 ± 0.138	−0.185 ± 0.113	2.11(17.65)	0.049	0.377 ± 0.179
Cz–C2	SMA (R)	EC_Soft_	9.21(1,20.25)	0.006	0.212 ± 0.167	−0.453 ± 0.141	3.03(20.25)	0.006	0.665 ± 0.219

Note: This table includes significant interaction effects, estimated mean changes from pre- to post-intervention within each group, and liner contrast results for HbR for different balance conditions in multiple channels. Channels are labeled using 10-10 coordinates, with specific anatomical landmarks and hemispheres (right and left) identified. Abbreviations: AG: Angular Gyrus; SMA: Supplementary Motor Area; STG: Superior Temporal Gyrus; PMC: Primary Motor Cortex; SMG: Supramarginal Gyrus; POP: Pars Opercularis; RA: Retrosubicular Area; S1: Primary Somatosensory Cortex. EO_Firm_: eyes open firm surface; EO_Soft_: eyes open soft surface; EC_Firm_: eyes closed firm surface; EC_Soft_: eyes closed soft surface; R = right hemisphere; L = left hemisphere; Post = post-intervention; Pre = pre-intervention; Δ = change; SE = standard errors; E = exercise group; Y = yoga group; and df: degrees of freedom.

**Table 5:** Significant simple (within-group) effects in HbO.

		Yoga				Exercise			
Channel (10-10)	Balance Condition	Mean Δ ± SE (Post − Pre)	F(df)	*p*	*d*_Cohen_	Mean Δ ± SE (Post − Pre)	F(df)	*p*	*d*_Cohen_
FC5–FC3	EO_Soft_	−0.353 ± 0.127	7.76(1,18.39)	0.012 *	0.81	0.058 ± 0.141	0.17(1,17.92)	0.686	0.13
FC1–FC3	EO_Soft_	−0.352 ± 0.111	10.04(1,20.30)	0.005 *	0.95	−0.122 ± 0.131	0.86(1,21.19)	0.364	0.32
CP2–CP4	EO_Soft_	−0.227 ± 0.088	6.63(1,18.56)	0.019 *	0.72	0.100 ± 0.102	0.95(1,19.02)	0.342	0.31
FC1–C1	EC_Firm_	0.334 ± 0.095	13.02(1,20.37)	0.002 *	1.08	0.219 ± 0.112	3.80(1,21.29)	0.065	0.68
C4–CP4	EC_Firm_	0.102 ± 0.115	0.78(1,18.32)	0.388	0.27	0.423 ± 0.142	8.81(1,19.02)	0.008 *	1.11
CP5–CP3	EC_Soft_	−0.092 ± 0.086	1.14(1,20.35)	0.297	0.27	0.355 ± 0.102	12.08(1,21.05)	0.002 *	1.01
P3–CP3	EC_Soft_	−0.236 ± 0.087	7.39(1,20.55)	0.013 *	0.085	0.172 ± 0.103	2.79(1,21.49)	0.109	0.61
C4–CP4	EC_Soft_	−0.060 ± 0.110	0.294(1,20.52)	0.594	0.17	0.405 ± 0.131	9.63(1,21.45)	0.005 *	1.10

Note: This table includes significant (in bold) and non-significant simple (i.e., within-groups) effects in HbO during different balance conditions in multiple channels. Channels are identified using the 10-10 system. Estimated mean changes (post − pre), standard errors (SE), and Cohen’s d are presented for each intervention group. * denotes significant difference after Bonferroni correction (*p* ≤ 0.025). Abbreviations: EO_Soft_: eyes open soft surface; EC_Firm_: eyes closed firm surface; EC_Soft_: eyes closed soft surface; Δ = change; SE: standard error; df: degrees of freedom; and *d*_Cohen_: Cohen’s d.

**Table 6:** Significant simple (within-group) effects in HbR.

		Yoga				Exercise			
Channel (10-10)	Balance Condition	Mean Δ ± SE (Post − Pre)	F(df)	*p*	*d*_Cohen_	Mean Δ ± SE (Post − Pre)	F(df)	*p*	*d*_Cohen_
Cz–C2	EO_Firm_	−0.436 ± 0.179	5.97(1,19.78)	0.024 *	0.81	0.225 ± 0.211	1.14(1,20.79)	0.297	0.41
FC2–FC4	EO_Firm_	−0.309 ± 0.123	6.31(1,20.50)	0.020 *	0.75	0.094 ± 0.145	0.42(1,21.40)	0.524	0.22
P4–CP4	EO_Firm_	−0.493 ± 0.141	12.14(1,20.09)	0.002 *	1.33	−0.166 ± 0.166	1.00(1,21.19)	0.329	0.44
CP5–CP3	EO_Soft_	0.023 ± 0.108	0.05(1,17.08)	0.831	0.05	0.418 ± 0.137	9.31(1,18.78)	0.007 *	0.94
T8–C6	EO_Soft_	−0.138 ± 0.117	1.40(1,19.88)	0.251	0.32	0.368 ± 0.139	7.05(1,20.68)	0.015 *	0.85
Cz–C2	EO_Soft_	−0.566 ± 0.178	10.11(1,20.28)	0.005 *	1.03	0.287 ± 0.210	1.87(1,21.27)	0.186	0.51
P4–CP4	EO_Soft_	−0.412 ± 0.140	8.65(1,20.08)	0.008 *	1.10	0.153 ± 0.164	0.86(1,21.18)	0.363	0.40
Cz–C2	EC_Firm_	−0.461 ± 0.136	11.47(1,10.04)	0.003 *	0.95	0.272 + 0.161	2.84(1,19.87)	0.107	0.55
CP6–CP4	EC_Firm_	−0.309 ± 0.125	6.11(1,20.16)	0.022 *	0.79	0.185 ± 0.148	1.58(1,21.14)	0.223	0.47
P4–CP4	EC_Firm_	−0.407 ± 0.139	8.51(1,20.69)	0.008 *	1.10	−0.028 ± 0.163	0.28(1,21.79)	0.868	0.07
C4–FC4	EC_Firm_	−0.263 ± 0.099	7.01(1,19.00)	0.016 *	0.63	−0.017 ± 0.125	0.19(1,19.68)	0.892	0.04
Cz–C2	EC_Soft_	−0.453 ± 0.141	10.27(1,19.73)	0.005 *	0.95	0.212 ± 0.167	1.60(1,20.62)	0.220	0.44

Note: This table includes significant (in bold) and non-significant simple (i.e., within-groups) effects in HbR during different balance conditions in multiple channels. Channels are identified using the 10-10 system. Estimated mean changes (post − pre), standard errors (SE), and Cohen’s d are presented for each intervention group. * denotes significant difference after Bonferroni correction (*p* ≤ 0.025). Abbreviations: EO_Firm_: eyes open firm surface; EO_Soft_: eyes open soft surface; EC_Firm_: eyes closed firm surface; EC_Soft_: eyes closed soft surface; Δ = change; SE: standard error; df: degrees of freedom; and *d*_Cohen_: Cohen’s d.

## Data Availability

The data presented in this study are available on request from the corresponding authors.

## References

[1] Brain Injury Association of America What Is the Difference between an Acquired Brain Injury and a Traumatic Brain Injury?. https://www.biausa.org/brain-injury/about-brain-injury/nbiic/what-is-the-difference-between-an-acquired-brain-injury-and-a-traumatic-brain-injury.

[2] Goldman L., Siddiqui E.M., Khan A., Jahan S., Rehman M.U., Mehan S., Sharma R., Budkin S., Kumar S.N., Sahu A. (2022). Understanding Acquired Brain Injury: A Review. Biomedicines.

[3] Langlois J.A., Rutland-Brown W., Wald M.M. (2006). The Epidemiology and Impact of Traumatic Brain Injury: A Brief Overview. J. Head Trauma Rehabil..

[4] Tsao C.W., Aday A.W., Almarzooq Z.I., Anderson C.A., Arora P., Avery C.L., Baker-Smith C.M., Beaton A.Z., Boehme A.K., Buxton A.E. (2023). Heart Disease and Stroke Statistics—2023 Update: A Report from the American Heart Association. Circulation.

[5] Hoofien D., Gilboa A., Vakil E., Donovick P.J. (2001). Traumatic Brain Injury (TBI) 10–20 Years Later: A Comprehensive Outcome Study of Psychiatric Symptomatology, Cognitive Abilities and Psychosocial Functioning. Brain Inj..

[6] Dikmen S.S., Machamer J.E., Powell J.M., Temkin N.R. (2003). Outcome 3 to 5 Years After Moderate to Severe Traumatic Brain Injury. Arch. Phys. Med. Rehabil..

[7] Whiteneck G.G., Cuthbert J.P., Corrigan J.D., Bogner J.A. (2016). Prevalence of Self-Reported Lifetime History of Traumatic Brain Injury and Associated Disability: A Statewide Population-Based Survey. J. Head Trauma Rehabil..

[8] Wilson L., Stewart W., Dams-O’Connor K., Diaz-Arrastia R., Horton L., Menon D.K., Polinder S. (2017). The Chronic and Evolving Neurological Consequences of Traumatic Brain Injury. Lancet Neurol..

[9] Ingersoll C.D., Armstrong C.W. (1992). The Effects of Closed-Head Injury on Postural Sway. Med. Sci. Sports Exerc..

[10] Johnson L., Williams G., Sherrington C., Pilli K., Chagpar S., Auchettl A., Beard J., Gill R., Vassallo G., Rushworth N. (2023). The Effect of Physical Activity on Health Outcomes in People with Moderate-to-Severe Traumatic Brain Injury: A Rapid Systematic Review with Meta-Analysis. BMC Public Health.

[11] Schmid A.A., Van Puymbroeck M., Altenburger P.A., Schalk N.L., Dierks T.A., Miller K.K., Damush T.M., Bravata D.M., Williams L.S. (2012). Poststroke Balance Improves With Yoga. Stroke.

[12] Stephens J.A., Van Puymbroeck M., Sample P.L., Schmid A.A. (2020). Yoga Improves Balance, Mobility, and Perceived Occupational Performance in Adults with Chronic Brain Injury: A Preliminary Investigation. Complement. Ther. Clin. Pract..

[13] Schmid A.A., Miller K.K., Van Puymbroeck M., Schalk N. (2016). Feasibility and Results of a Case Study of Yoga to Improve Physical Functioning in People with Chronic Traumatic Brain Injury. Disabil. Rehabil..

[14] Gothe N.P., Khan I., Hayes J., Erlenbach E., Damoiseaux J.S. (2019). Yoga Effects on Brain Health: A Systematic Review of the Current Literature. Brain Plast..

[15] Gothe N.P., Hayes J.M., Temali C., Damoiseaux J.S. (2018). Differences in Brain Structure and Function Among Yoga Practitioners and Controls. Front. Integr. Neurosci..

[16] Surgent O.J., Dadalko O.I., Pickett K.A., Travers B.G. (2019). Balance and the Brain: A Review of Structural Brain Correlates of Postural Balance and Balance Training in Humans. Gait Posture.

[17] Dijkstra B.W., Bekkers E.M.J., Gilat M., De Rond V., Hardwick R.M., Nieuwboer A. (2020). Functional Neuroimaging of Human Postural Control: A Systematic Review with Meta-Analysis. Neurosci. Biobehav. Rev..

[18] Ouchi Y., Okada H., Yoshikawa E., Nobezawa S., Futatsubashi M. (1999). Brain Activation during Maintenance of Standing Postures in Humans. Brain.

[19] Ouchi Y., Okada H., Yoshikawa E., Futatsubashi M., Nobezawa S. (2021). Absolute Changes in Regional Cerebral Blood Flow in Association with Upright Posture in Humans: An Orthostatic PET Study. J. Nucl. Med..

[20] Pinti P., Aichelburg C., Gilbert S., Hamilton A., Hirsch J., Burgess P., Tachtsidis I. (2018). A Review on the Use of Wearable Functional Near-Infrared Spectroscopy in Naturalistic Environments. Jpn. Psychol. Res..

[21] Pinti P., Tachtsidis I., Hamilton A., Hirsch J., Aichelburg C., Gilbert S., Burgess P.W. (2020). The Present and Future Use of Functional Near-infrared Spectroscopy (fNIRS) for Cognitive Neuroscience. Ann. N. Y. Acad. Sci..

[22] Scholkmann F., Kleiser S., Metz A.J., Zimmermann R., Mata Pavia J., Wolf U., Wolf M. (2014). A Review on Continuous Wave Functional Near-Infrared Spectroscopy and Imaging Instrumentation and Methodology. NeuroImage.

[23] Stephens J.A., Press D., Atkins J., Duffy J.R., Thomas M.L., Weaver J.A., Schmid A.A. (2023). Feasibility of Acquiring Neuroimaging Data from Adults with Acquired Brain Injuries before and after a Yoga Intervention. Brain Sci..

[24] Herold F., Wiegel P., Scholkmann F., Thiers A., Hamacher D., Schega L. (2017). Functional Near-Infrared Spectroscopy in Movement Science: A Systematic Review on Cortical Activity in Postural and Walking Tasks. Neurophoton.

[25] Mihara M., Miyai I., Hattori N., Hatakenaka M., Yagura H., Kawano T., Kubota K. (2012). Cortical Control of Postural Balance in Patients with Hemiplegic Stroke. NeuroReport.

[26] Helmich I., Berger A., Lausberg H. (2016). Neural Control of Posture in Individuals with Persisting Postconcussion Symptoms. Med. Sci. Sports Exerc..

[27] Fujimoto H., Mihara M., Hattori N., Hatakenaka M., Kawano T., Yagura H., Miyai I., Mochizuki H. (2014). Cortical Changes Underlying Balance Recovery in Patients with Hemiplegic Stroke. NeuroImage.

[28] Leach H.J., Hidde M.C., Portz J.D., Van Puymbroeck M., Sharp J.L., Fox A.L., Schmid A.A., Fruhauf C.A. (2023). Matching Exercise Volume in Active Control Groups for Yoga Interventions. Altern. Ther. Health Med..

[29] Stephens J.A., Hernandez-Sarabia J.A., Sharp J.L., Leach H.J., Bell C., Thomas M.L., Buryznska A.Z., Weaver J.A., Schmid A.A. (2023). Adaptive Yoga versus Low-Impact Exercise for Adults with Chronic Acquired Brain Injury: A Pilot Randomized Control Trial Protocol. Front. Hum. Neurosci..

[30] King P.R., Donnelly K.T., Donnelly J.P., Dunnam M., Warner G., Kittleson C.J., Bradshaw C.B., Alt M., Meier S.T. (2012). Psychometric Study of the Neurobehavioral Symptom Inventory. J. Rehabil. Res. Dev..

[31] Goble D.J., Khan E., Baweja H.S., O’Connor S.M. (2018). A Point of Application Study to Determine the Accuracy, Precision and Reliability of a Low-Cost Balance Plate for Center of Pressure Measurement. J. Biomech..

[32] O’Connor S.M., Baweja H.S., Goble D.J. (2016). Validating the BTrackS Balance Plate as a Low Cost Alternative for the Measurement of Sway-Induced Center of Pressure. J. Biomech..

[33] Richmond S.B., Dames K.D., Goble D.J., Fling B.W. (2018). Leveling the Playing Field: Evaluation of a Portable Instrument for Quantifying Balance Performance. J. Biomech..

[34] Zimeo Morais G.A., Balardin J.B., Sato J.R. (2018). fNIRS Optodes’ Location Decider (fOLD): A Toolbox for Probe Arrangement Guided by Brain Regions-of-Interest. Sci. Rep..

[35] Brigadoi S., Cooper R.J. (2015). How Short Is Short? Optimum Source–Detector Distance for Short-Separation Channels in Functional near-Infrared Spectroscopy. NPh.

[36] Tachtsidis I., Scholkmann F. (2016). False Positives and False Negatives in Functional Near-Infrared Spectroscopy: Issues, Challenges, and the Way Forward. Neurophoton.

[37] Peirce J., Gray J.R., Simpson S., MacAskill M., Höchenberger R., Sogo H., Kastman E., Lindeløv J.K. (2019). PsychoPy2: Experiments in Behavior Made Easy. Behav. Res..

[38] Peirce J.W. (2007). PsychoPy—Psychophysics Software in Python. J. Neurosci. Methods.

[39] Peirce J.W. (2008). Generating Stimuli for Neuroscience Using PsychoPy. Front. Neuroinform..

[40] Goble D.J., Brown E.C., Marks C.R.C., Baweja H.S. (2020). Expanded Normative Data for the Balance Tracking System Modified Clinical Test of Sensory Integration and Balance Protocol. Med. Devices Sens..

[41] Prieto T.E., Myklebust J.B., Hoffmann R.G., Lovett E.G., Myklebust B.M. (1996). Measures of Postural Steadiness: Differences between Healthy Young and Elderly Adults. IEEE Trans. Biomed. Eng..

[42] Baker W.B., Parthasarathy A.B., Busch D.R., Mesquita R.C., Greenberg J.H., Yodh A.G. (2014). Modified Beer-Lambert Law for Blood Flow. Biomed. Opt. Express.

[43] Fishburn F.A., Ludlum R.S., Vaidya C.J., Medvedev A.V. (2019). Temporal Derivative Distribution Repair (TDDR): A Motion Correction Method for fNIRS. NeuroImage.

[44] Plichta M.M., Herrmann M.J., Baehne C.G., Ehlis A.-C., Richter M.M., Pauli P., Fallgatter A.J. (2007). Event-Related Functional near-Infrared Spectroscopy (fNIRS) Based on Craniocerebral Correlations: Reproducibility of Activation?. Hum. Brain Mapp..

[45] Pollonini L., Olds C., Abaya H., Bortfeld H., Beauchamp M.S., Oghalai J.S. (2014). Auditory Cortex Activation to Natural Speech and Simulated Cochlear Implant Speech Measured with Functional Near-Infrared Spectroscopy. Hear. Res..

[46] Ainsworth B.E., Haskell W.L., Herrmann S.D., Meckes N., Bassett D.R.J., Tudor-Locke C., Greer J.L., Vezina J., Whitt-Glover M.C., Leon A.S. (2011). 2011 Compendium of Physical Activities: A Second Update of Codes and MET Values. Med. Sci. Sports Exerc..

[47] Borg G. (1998). Borg’s Perceived Exertion and Pain Scales.

[48] Wang Y., Yan J., Wen J., Yu T., Li X. (2016). An Intracranial Electroencephalography (iEEG) Brain Function Mapping Tool with an Application to Epilepsy Surgery Evaluation. Front. Neuroinform..

[49] Kelley K., Preacher K.J. (2012). On Effect Size. Psychol. Methods.

[50] Rosenthal J.A. (1996). Qualitative Descriptors of Strength of Association and Effect Size. J. Soc. Serv. Res..

[51] Berg K., Wood-Dauphinee S., Williams J. (1995). The Balance Scale: Reliability Assessment with Elderly Residents and Patients with an Acute Stroke. Scand. J. Rehabil. Med..

[52] Hyndman D., Ashburn A., Yardley L., Stack E. (2006). Interference between Balance, Gait and Cognitive Task Performance among People with Stroke Living in the Community. Disabil. Rehabil..

[53] Palmisano S., Fasotti L., Bertens D. (2020). Neurobehavioral Initiation and Motivation Problems After Acquired Brain Injury. Front. Neurol..

[54] Strotzer M. (2009). One Century of Brain Mapping Using Brodmann Areas. Clin. Neuroradiol..

[55] Takakura H., Nishijo H., Ishikawa A., Shojaku H. (2015). Cerebral Hemodynamic Responses During Dynamic Posturography: Analysis with a Multichannel Near-Infrared Spectroscopy System. Front. Hum. Neurosci..

[56] Clower D.M., West R.A., Lynch J.C., Strick P.L. (2001). The Inferior Parietal Lobule Is the Target of Output from the Superior Colliculus, Hippocampus, and Cerebellum. J. Neurosci..

[57] Kantak S.S., Stinear J.W., Buch E.R., Cohen L.G. (2012). Rewiring the Brain: Potential Role of the Premotor Cortex in Motor Control, Learning, and Recovery of Function Following Brain Injury. Neurorehabil. Neural Repair.

[58] Nachev P., Kennard C., Husain M. (2008). Functional Role of the Supplementary and Pre-Supplementary Motor Areas. Nat. Rev. Neurosci..

[59] Karim H., Fuhrman S.I., Sparto P., Furman J., Huppert T. (2013). Functional Brain Imaging of Multi-Sensory Vestibular Processing during Computerized Dynamic Posturography Using near-Infrared Spectroscopy. NeuroImage.

[60] Jahn K., Deutschländer A., Stephan T., Kalla R., Wiesmann M., Strupp M., Brandt T. (2008). Imaging Human Supraspinal Locomotor Centers in Brainstem and Cerebellum. NeuroImage.

[61] Tomaiuolo F., MacDonald J.D., Caramanos Z., Posner G., Chiavaras M., Evans A.C., Petrides M. (1999). Morphology, Morphometry and Probability Mapping of the Pars Opercularis of the Inferior Frontal Gyrus: An in Vivo MRI Analysis. Eur. J. Neurosci..

[62] Lobel E., Kleine J.F., Bihan D.L., Leroy-Willig A., Berthoz A. (1998). Functional MRI of Galvanic Vestibular Stimulation. J. Neurophysiol..

